# Development of adaptability of foreign breeds of water buffalo in Philippine tropical climate

**DOI:** 10.1093/af/vfad041

**Published:** 2023-10-13

**Authors:** Excel Rio S Maylem, Gerald E Ramos, Shanemae M Rivera, Edwin C Atabay, Eufrocina P Atabay

**Affiliations:** Reproduction and Physiology Section, Philippine Carabao Center National Headquarters, Science City of Munoz, Nueva Ecija, Philippines, 3120; Reproduction and Physiology Section, Philippine Carabao Center National Headquarters, Science City of Munoz, Nueva Ecija, Philippines, 3120; Reproduction and Physiology Section, Philippine Carabao Center National Headquarters, Science City of Munoz, Nueva Ecija, Philippines, 3120; Reproduction and Physiology Section, Philippine Carabao Center National Headquarters, Science City of Munoz, Nueva Ecija, Philippines, 3120; Reproduction and Physiology Section, Philippine Carabao Center National Headquarters, Science City of Munoz, Nueva Ecija, Philippines, 3120

**Implications**

- HSP70 gene expression is an excellent biomarker for heat stress and thermotolerance.
- Philippine Native Water Buffalo is more adaptive to heat stress, hence, highly thermotolerant.
- Foreign breeds of buffaloes moved into the Philippines have already developed adaptability to the tropical climate.
- Thermotolerant and productive buffalo breeds should be a priority in breeding and genetic selection for improved animal production.

## Introduction

Heat stress disturbs the body’s normal behavioral, immunological, and physiological functions through direct and indirect actions facilitated by alterations in energy balance. Overall, heat stress impairs animal productivity.

Indigenous and native livestock breeds are the hardiest due to their ability to cope and remain productive in harsh environments. Hence, different breeds have different adaptive potentials. Thermotolerance is primarily owed to physiological and genetic adaptations. Domestic buffaloes in Brazil are naturally selected for adaptation to local climatic conditions such as high temperature and humidity and high direct solar radiation. In contrast, breeds of buffaloes from India and southern Asia can adapt to hot, humid areas of muddy and swampy lands. Albeit heat stress, native breeds maintain their reproductive potential since they have smaller body sizes than larger and exotic breeds with bigger bodies and higher energy requirements.

Using technological devices to monitor animals’ responses to extreme heat can help increase productivity efficiency and contribute to comfort and welfare. Further, genetic selection and breeding heat tolerant to highly productive breeds have gained importance worldwide. Thus, aside from physiological responses, thermotolerant genetic markers are essential in identifying adaptability for future productivity.

In the Philippines, water buffalo farmers are highly aware of the climate change impacts on their animals and are doing small adaptation strategies to sustain production and maintain their income (Escarcha et al., 2018). Thus, efforts should be developed to produce thermotolerant breeds of buffaloes for climate-resilient animal production. Therefore, the adaptability of foreign breeds of buffaloes in different environmental conditions is inevitable to maintain their productivity and overall welfare.

## Use of HSP70 as a Thermotolerance Marker

HSPs are highly conserved proteins induced by elevated temperatures or different cellular stresses. When HSP70 expression escalates, it indicates adaptation to extreme environmental stresses, hence, its significant role in cellular thermotolerance. Though stress-induced HSP expression represents the chaperonin action in the cell to protect it, individual animals and breeds still differ in their capacity to manage stress. Hence, the use of HSP70 as a thermotolerance indicator and adaptability to improve animal reproduction activities and other physiological systems affected by the challenging environment.

## Thermotolerance and Adaptability of Foreign and Native breeds

### Physiological response

Ruminants’ physiological responses have long been used to determine their reactions to heat stress, including pulse, rectal and sweating rates, and skin temperature. Usually, higher respiration rates and sweating rates are observed when animals are experiencing an increase in temperature. Although influential in determining the severity of heat stress, there is still a genetic variation that can be attributed to the differences in the response mechanisms. Several studies present data to suggest that physiological adaptability varies among indigenous, cross-bred, and purebred animals, with indigenous livestock showcasing less physiological variability. These findings hold in our research, where foreign and Philippine Native breeds of buffaloes presented a significantly different pulse rate (*P* < 0.001) but not rectal rate and rectal temperature (*P* > 0.001) during extreme environmental temperatures. Similarly, Tarai buffalo rectal rate is used as a physiological characteristic and a marker for heat stress (Manjari et al., 2015). Tarai breeds presented a correlation between HSP70 gene expression and rectal rate, pulse rate, and rectal temperature from (r = 0.86 to 0.98), while Philippine Native and foreign breeds (Italian, Brazilian, and Bulgarian) in the country did not show a significant correlation (*P* > 0.001). Thus, breed and genetic variation have something to do with the different physiological responses of the animals. In addition, since rectal rate and rectal temperature are not significantly different among the different breeds living in the country for 5 to 8 yr, they have already developed adaptability to heat stress conditions present.

### Cellular and molecular response

**Figure 1.**
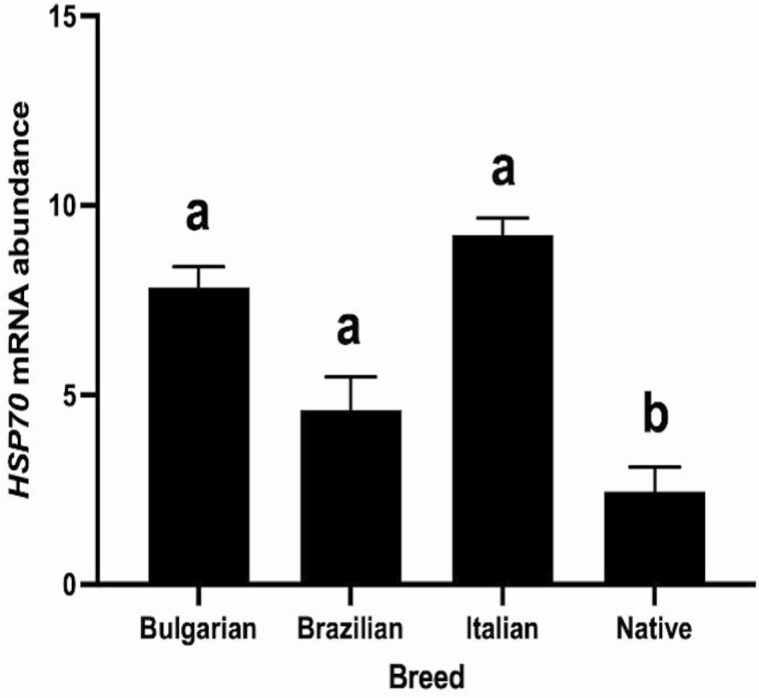
HSP70 gene expression of bulls from different breeds. Bars without a common letter differ (*P* < 0.05; Blood sample was collected once a month for 1 yr).

HSP70 has been used as a marker of heat stress in livestock animals, with indigenous breeds having fewer HSPs because of their thermotolerant ability. In the present study, RNA was extracted from the lymphocytes of blood collected monthly for 1 yr from Native and imported foreign breeds of buffaloes in the Philippines. Animals did not receive any treatment during the entire collection. It was found that foreign breeds’ HSP70 gene expression is significantly (*P* < 0.05) higher than the Native breed (Figure 1). With only a 3.8-fold increase in HSP70 gene expression of foreign breeds, we could infer that the foreign breeds, though stressed, have developed adaptability in the present environmental condition. These variations in the expression of the HSP70 gene in blood lymphocytes can also be attributed to the genetic structure of each breed. The degree or expression of HSP70, with its chaperonin function, explains an animal’s tolerance to heat stress and its ability to cope, survive and eventually adapt to the present environmental condition. Similar to the Tarai buffalo, which is native to India, HSP70, a cellular marker for heat and humidity stress, has a 2.37 ± 0.12 gene expression during summer and 0.29 ± 0.04 during winter (Archana et al., 2017). The buffaloes here in the Philippines, regardless of the breed, have an average of 7.63 HSP70 gene expression during the hot-dry season compared to 4.43 during the cold-wet season. Thus, the Philippine Native breed is highly thermotolerant, while foreign breeds can adapt to and thrive in the environmental condition of the tropical country.

With physiological responses and HSP70 gene expression both effective in determining heat stress across all breeds, it could substantiate the use of HSP70 as a biomarker for thermotolerance in water buffaloes.

### Effects on semen quality

Semen quality was observed in different breeds of buffaloes in the country. It was found that there is no significant difference (*P* > 0.001) between the semen quality (initial motility and sperm concentration) of the Native breed to the foreign breeds. Although foreign breeds are subjected to thermal stress, as seen in their physiological responses, and HSP70 gene expression, the semen quality was unaffected; this is attributed to the chaperonin mechanism of the HSP70 to the cells, alleviating heat stress experienced and the adaptability of the foreign breeds to the tropical country. Hence, semen production is maintained and less likely a problem for imported breeds once they get adapted to the environmental condition in the location where they moved.

## Conclusion

The Philippine Water Buffalo is highly thermotolerant based on its HSP70 gene expression and physiological responses, which causes its ability to maintain good semen quality. In contrast, foreign breeds are more heat stressed in present environmental conditions with higher HSP70 gene expression. However, foreign breeds have adapted to the present environmental condition as seen in their physiological responses comparable to Native breeds with even better semen quality. The chaperonin effect from HSP70 has served the foreign breeds well, and their adaptability is presented. Thus, imported foreign breeds can thrive in tropical countries like the Philippines. Therefore, the goal of crossing native breeds to imported breeds to produce thermotolerant and highly productive buffaloes should be implemented as part of the precision livestock farming dedicated to increasing agricultural food production and overall economic gain in the country and even worldwide.

## About the Author(s)

**Dr. Excel Rio S. Maylem** is a Senior Science Research Specialist at the Reproduction and Physiology Section, Philippine Carabao Center, National Headquarters and Genepool, Science City of Munoz, Nueva Ecija. She is also an affiliate professor at the Department of Biological Sciences, College of Sciences, Central Luzon State University, Science City of Munoz, Nueva Ecija. She finished her PhD in Animal Science major in Reproduction and Physiology, Oklahoma State University, Stillwater, OK, USA under the Fulbright Graduate Degree Program and has served as a Graduate Research and Teaching Assistant in the Department of Animal and Food Sciences. Her Masters and Bachelor’s Degree in Biology is in Central Luzon State University. Her research interests are on ovarian function, molecular reproduction, assisted reproductive techniques and stress physiology.

**Gerald E. Ramos** is currently working at a Dairy Farm Herdsman, Stella Dairy, Roelands, Western Australia. He served as a researcher in Philippine Carabao Center, National Headquarters and Genepool, Science City of Munoz, Nueva Ecija focusing on HSP70s in water buffalo blood and semen. His other research interests are large ruminant reproduction and nutrition, animal physiology. Mr. Ramos finished his Bachelor’s Degree in Agriculture major in Animal Science, Central Luzon State University, Science City of Munoz, Nueva Ecija.

**Shanemae M. Rivera** is currently working as a research specialist for the Integrated Protein Research and Development Center of the Ateneo de Manila University. She is an MSc in Agricultural Biotechnology graduate from the Hungarian University of Agriculture and Life Sciences. For many years, she worked for the Philippine Carabao Center with other authors and studied the correlation among Heat Shock Protein 70, seasonal changes, and heat tolerance in water buffaloes. At the moment, she is working with other young researchers in producing high-quality, socio-medically important recombinant proteins for rapid testing and diagnostics.

**Edwin C. Atabay** is a Scientist II, Scientific Career System, Senior Science Research Specialist and Chief, Operations of the Philippine Carabao Center, Science City of Munoz, Nueva Ecija. He finished his postdoctoral degree through the Fulbright-Philippine Agriculture Scholarship Program (FPASP) for Advance Research Awards, University of Tennessee, Knoxville, USA and as a research fellow of the DA-Biotech Program, Department of Agriculture, University of Connecticut, USA. His PhD is in Veterinary Medicine (Theriogenology), Hokkaido University, Sapporo, Japan, Masters of Science Master of Science in Animal Science, Central Luzon State University, Nueva Ecija. Dr. Atabay is also a Veterinarian by profession. His research interests are on Animal Reproductive Biotechnology and Physiology (Cryopreservation of Male and Female Gametes and Somatic Cells, in vitro Fertilization and Cloning in Large Animals).

**Dr. Eufrocina P. Atabay** is a Scientist II, Scientific Career System, Supervising Science Research Specialist and Focal Person, Intellectual Property and Technology Transfer, Philippine Carabao Center. Her postdoctoral degree was from University of Massachusetts, Amherst, USA as a Senior Scholar, FPASP for Advance Research. She also did a research fellowship in the Department of Agriculture and University of Connecticut, USA under the DA-Biotech Program. She finished her Doctor of Philosophy in Veterinary Medicine (Theriogenology) in Hokkaido University, Sapporo, Japan, while her Masters of Science in Animal Science from University of the Philippines, Los Banos, Laguna. Dr. Atabay is also a licensed Veterinarian from Central Luzon State University, Science City of Munoz, Nueva Ecija. Her research interests are in Animal Reproductive Biotechnology and Physiology (Cryopreservation of Male and Female Gametes and Somatic Cells, in vitro Fertilization and Cloning in Large Animals).
